# Imaging of Fluoride Ion in Living Cells and Tissues with a Two-Photon Ratiometric Fluorescence Probe

**DOI:** 10.3390/s150101611

**Published:** 2015-01-14

**Authors:** Xinyue Zhu, Jianxi Wang, Jianjian Zhang, Zhenjie Chen, Haixia Zhang, Xiaoyu Zhang

**Affiliations:** 1 State Key Laboratory of Applied Organic Chemistry, College of Chemistry and Chemical Engineering, Lanzhou University, Lanzhou 730000, China; E-Mails: zhuxy12@lzu.edu.cn (X.Z.); wangjx13@lzu.edu.cn (J.W.); zhangjj13@lzu.edu.cn (J.Z.); 2 Institute of Physiology, School of Basic Medical Sciences, Lanzhou University, Lanzhou 730000, China; E-Mails: chenzj12@lzu.edu.cn (Z.C.); zhangxyu@lzu.edu.cn (X.Z.)

**Keywords:** two-photon excited, ratiometric fluorescence, imaging, fluoride

## Abstract

A reaction-based two-photon (TP) ratiometric fluorescence probe **Z2** has been developed and successfully applied to detect and image fluoride ion in living cells and tissues. The **Z2** probe was designed designed to utilize an ICT mechanism between *n*-butylnaphthalimide as a fluorophore and *tert*-butyldiphenylsilane (TBDPS) as a response group. Upon addition of fluoride ion, the Si-O bond in the **Z2** would be cleaved, and then a stronger electron-donating group was released. The fluorescent changes at 450 and 540 nm, respectively, made it possible to achieve ratiometric fluorescence detection. The results indicated that the **Z2** could ratiometrically detect and image fluoride ion in living cells and tissues in a depth of 250 μm by two-photon microscopy (TPM).

## 1. Introduction

Fluoride anion as an essential element of human body plays significant roles in chemical and biological processes [1–3], which is widely added to toothpastes, pharmaceutical agents and drinking water due to the beneficial effects for preventing dental caries [4], enamel demineralization while wearing orthodontic appliances and treatment of osteoporosis [5,6]. However, excessive intake of fluoride ion may result in skeletal fluorosis [7–9], urolithiasis [10], kidney failure [11], or even cancer [12]. Moreover, sodium fluoride (NaF) is associated with various cell signal components, and can induce apoptosis at a higher concentration in mammalian cells [13,14]. Hence, it is very important to develop an efficient method to monitor quantitatively fluoride anion, particularly NaF in biological systems. Among the analytical methods developed so far [15–18], fluorescence imaging has been proved to the most practical one owing to the simplicity, specificity and sensitivity. However, most of the reported fluorescence probes for detecting fluoride ion were excited by one-photon (OP) [19–31]. To imaging fluoride ion in biological systems, TP fluorescence probe would be the much better choice due to the deeper penetration depth, lower tissue auto-fluorescence and self-absorption, reduced photobleaching and photodamage, and high spatial resolution [32–38]. Up to now, very few TP fluorescent probes for imaging fluoride anion in biological matrices have been developed [39,40].

Considering these facts, we developed a reaction-based two-photon ratiometric fluorescence **Z2** to detect and image fluoride ion in this study. The ratiometric fluorescence strategy offered more reliable quantitative measurement results compared with conventional fluorescence turn-on probes, which were affected seriously by the experimental conditions.

The TP ratiometric fluorescence **Z2** was designed according to an internal charge transfer (ICT) mechanism utilizing *n*-butylnaphthalimide as a fluorophore owing to its good photostability, large Stokes shift and excellent ICT structure, and TBDPS as a response group due to the high affinity between F^−^ and silicon [40]. Upon addition of fluoride ion, The Si-O bond of the **Z2** probe would be cleaved to release a stronger electron-donating group, which could greatly stabilize the charge-transfer excited state of naphthalimide fluorophore to reveal a larger Stokes shift, thereby, achieving ratiometric fluorescence detection. As expected, the results showed that the **Z2** could ratiometrically detect and image fluoride ion in living cells and tissues in a depth of 250 μm by two-photon microscopy (TPM).

## 2. Experimental Section

### 2.1. Cell Culture

HeLa cells were obtained from the biomedical engineering center of Hunan University (Changsha, China). The cells were propagated in Dulbecco's Modified Eagle Medium (DMEM) supplemented with 10% (v/v) fetal bovine serum, penicillin (100 μg/mL), and streptomycin (100 μg/mL). Cells were maintained under a humidified atmosphere of 5% CO_2_ and at 37 °C incubator. For cell imaging studies, cells were seeded into a confocal dish and incubated at 37 °C in a CO_2_ incubator for one day. Then the cells were washed for three times with PBS buffer (pH 7.4) and divided into three groups. The first group was used as a reference and the **Z2** probe was added to the second gorup, and finally in the third group, the cells were treated with **Z2** and fluoride (15 μM) for 20 min. At last all of these cells were washed with PBS buffer for three times to remove the free compounds before analysis. The treatments illustrated above were operated in DMEM at 37 °C in a CO_2_ incubator.

### 2.2. Tissue Culture

Tissue slices were prepared from Hela cells. A total of 2 × 10^6^ Hela cells diluted in 100 μL of serum-free PBS medium were injected subcutaneously into the right flank of 6- to 8-week-old BALB/c-nude mice to inoculate tumors. After Hela cells inoculation was for 15 days, mice were sacrificed. Tumors were transferred and embedded with O.C.T (Sakura Finetek, USA, Torrance, CA, USA) for preparing frozen sections. The tissues were cut into 400 μm- thick slices using a vibrating-blade microtome. The slices were incubated with 50 μM of **Z2** for 12 h at 4 °C. After washing with PBS for three times, the slices were mounted with 10% glycerol and sealed with nail varnish on a glass substrate.

### 2.3. Measurement of Two-Photon Cross Section

The two-photon cross section (δ) was determined using a femtosecond (fs) fluorescence measurement technique [41]. To measure δ of **Z2** and the reaction product (**1**) of **Z2** (5.0 × 10^−6^ M) with F^−^ (1.0 × 10^−4^ M), the reaction mixture dissolved in 20 mM HEPES (30% DMSO, pH 7.4) was kept at 25 °C for 1 h before the measurement. The two-photon induced fluorescence intensity was measured at 700–880 nm using Rhodamine 6G as the reference, whose two-photon property has been well characterized in the literature [42]. The intensities of the two-photon induced fluorescence spectra of the reference and the samples emitted at the same excitation wavelength were determined. The TPA cross section was calculated according to Equation (1):

(1) $$\delta_{s}=\delta_{r}\frac{\phi_{r}}{\phi_{s}}\frac{C_{r}}{C_{s}}\frac{n_{r}}{n_{s}}\frac{S_{s}}{S_{r}}$$

The subscripts *s* and *r* stand for the sample and reference, respectively; δ is the TPA cross sectional value, φ as the fluorescence quantum yield and *n* as the refractive index of the solvents; *C* is the concentration and *S* as the intensity of two-photon fluorescence emission.

### 2.4. Fluorescence Imaging

Two-photon fluorescence images of the dye labeled cells and tissues were obtained by exciting the probes with a modelocked titanium-sapphire laser source (Mai Tai DeepSee, Spectra-Physics, Irvine, CA, USA, 80 MHz, 90 fs) set at wavelength 780 nm with FV1000 laser confocal microscope I × 81 (Olympus, Tokyo, Japan) with 20 objective, numerical aperture (NA) = 0.4. The image signals in the 500–600 nm range were collected by internal PMTs in a 12 bit unsigned 1024 × 1024 pixels image at 40 Hz scan speed.

### 2.5. Instruments and Materials

^1^H-NMR and ^13^C-NMR spectra were recorded on a JEOL-ECS-400MHz (JEOL Ltd., Tokyo, Japan) using tetramethylsilane (TMS) as an internal standard. Mass spectra were obtained by a Thermo LTQ Orbitrap XL Mass spectrophotometer (Thermo Fisher Scientific Inc., Waltham, MA, USA) and a LQC system (Finngan MAT, San Jose, CA, USA). UV-Vis absorption spectra were recorded on a TU-1810 spectrophotometer (PEXI, Beijing, China). The OP excited fluorescence spectra were measured out on a RF-5310PC spectrofluorophotometer (Shimadzu, Tokyo, Japan) and TP excited fluorescence spectra and the fluorescent quantum yield were determined on a FLSP920 fluorescence spectrometer (Edinburgh Instruments Ltd., Livingston, UK). TP fluorescence images were recorded by an Olympus FV1000 laser confocal microscope. The pH values were calibrated with a model 868 pH meter (Leici, Shanghai, China). Unless otherwise noted, materials from commercial suppliers were used without further purification.

### 2.6. Synthesis and Characteristic of **Z2**

#### 2.6.1. Synthesis of *N*-Butyl-4-Br-1,8-naphthalimide (**3**)

To a solution of 4-Br-1, 8-naphthalic anhydride (2.77 g, 10.0 mmol) in C_2_H_5_OH (50 mL) was added 1-aminobutane (0.88 g, 12.0 mmol). The mixture was stirred under reflux in a N_2_ atmosphere for 6 h. After cooling down to room temperature, the solvent was removed *in vacuo*, and the crude product was purified by silica gel column chromatography (CH_2_Cl_2_) to give 2.52 g of **3** (75%). ^1^H-NMR (CDCl_3_) δ 8.57 (dd, *J* = 7.3, 0.84 Hz, 1 H), 8.46 (dd, *J* = 8.44, 1.04 Hz, 1 H), 8.32 (d, *J* = 7.8 Hz, 1 H), 7.95 (d, *J* = 7.8 Hz, 1 H), 7.77 (dd, *J* = 4.1, 7.4 Hz, 1 H), 4.13 (t, *J* = 7.6 Hz, 2 H), 1.73–1.63 (m, 2 H), 1.48−1.37 (m, 2 H), 0.96 (t, *J* = 7.4 Hz, 3 H) ppm; ^13^C-NMR (CDCl_3_) δ 163.0 (2C), 133.2, 132.0, 131.2, 131.1, 130.5, 130.2, 128.9, 128.1, 123.1, 122.2, 40.4, 30.2, 20.5, 14.0 ppm. MS (ESI): [M + H]^+^ 332.2035.

#### 2.6.2. Synthesis of *N*-Butyl-4-methoxy-1,8-naphthalimide (**2**)

K_2_CO_3_ (1.66 g, 18.0 mmol) was added into a solution of **3** (1.99 g, 6.0 mmol) in CH_3_OH (40 mL). The mixture was stirred under reflux in a N_2_ atmosphere for 24 h. After cooling down to room temperature, the solvent was removed *in vacuo*, and the crude product was filtered and washed by H_2_O to give **2** (1.36 g, 80%). ^1^H-NMR (CDCl_3_) δ 8.57 (dd, *J* = 7.3, 1.2 Hz, 1 H), 8.52 (dd, *J* = 8.2, 1.8 Hz, 2 H), 7.68 (dd, *J* = 8.4, 7.4 Hz, 1 H), 7.02 (d, *J* = 7.3, 8.3 Hz, 1 H), 4.16 (t, *J* = 7.5 Hz, 2 H), 4.11 (s, 3 H), 1.74−1.65 (m, 2 H), 1.49−1.38 (m, 2 H), 0.97 (t, *Ј* = 7.4 Hz, 3 H) ppm; ^13^C-NMR (CDCl_3_) δ 164.5, 163.9, 160.7, 133.3, 131.4, 129.3, 128.5, 125.9, 123.4, 122.4, 115.1, 105.1, 56.1, 40.1, 30.2, 20.4, 13.8 ppm. MS (ESI): [M + H]^+^ 284.1283.

#### 2.6.3. Synthesis of *N*-Butyl-4-hydroxy-1,8-naphthalimide (**1**)

Compound **2** (1.14 g, 4.0 mmol) was dissolved in HI solution (57%, 50 mL), and then the mixture was stirred under reflux in a N_2_ atmosphere for 7 h. After cooling down to room temperature, the solution was adjusted to a neutral pH by a Na_2_CO_3_ saturated solution. Then the precipitate was filtered and washed by H_2_O to give **1** (0.86 g, 80%). ^1^H-NMR (DMSO-d_6_) δ 11.88 (s, 1 H), 8.53 (d, *J* = 7.6 Hz, 1 H), 8.48 (d, *J* = 7.0 Hz, 1 H), 8.36 (d, *J* = 8.2 Hz, 1 H), 7.77 (t, *J* = 7.7 Hz, 1 H), 7.16 (d, *J* = 8.2 Hz, 1 H), 4.02 (t, *J* = 7.2 Hz, 2 H), 1.63−1.54 (m, 2 H), 1.39−1.27 (m, 2 H), 0.92 (t, *J* = 7.3 Hz, 3 H) ppm; ^13^C-NMR (DMSO-d_6_) δ 164.2, 163.5, 160.7, 134.0, 131.6, 130.0, 129.4, 126.1, 122.8, 122.3, 113.1, 110.4, 40.2, 30.3, 20.3, 14.3 ppm. MS (ESI): [M + H]^+^ 270.0521.

#### 2.6.4. Synthesis of **Z2**

A solution of **1** (0.81 g, 3.0 mmol) in *N*, *N*-dimethylformamide (DMF, 50 mL) was added with imidazole (0.18 g, 3.3 mmol). After the mixture was stirred under N_2_ atmosphere for 15 min, *tert*-butyldiphenylsilyl chloride (TBDPSCl) (78 μL, 3.3 mmol) was added dropwise to the mixture. After the reaction mixture was stirred at room temperature for 6 h, the solvent was removed *in vacuo*, and the crude product was recrystallized from CH_2_Cl_2_ to give **Z2** (1.06 g, 70%). ^1^H-NMR (CDCl_3_, shown in Figure S1) δ 8.78 (d, *J* = 8.3Hz, 1 H), 8.65 (d, *J* = 7.3Hz, 1 H), 8.16 (d, *J* = 8.2 Hz, 1 H), 7.82−7.70 (m, 5 H), 7.48 (d, *J* = 7.3 Hz, 1 H), 7.46−7.36 (m, 5 H), 6.62 (d, *J* = 8.2 Hz, 1 H), 4.14 (t, *J* = 7.5 Hz, 2 H), 1.73−1.63 (m, 2 H), 1.47-1.36 (m, 2 H), 1.21 (s, 9 H), 0.95 (t, *J* = 7.4 Hz, 3 H) ppm; ^13^C- NMR (shown in Figure S2) δ 164.6, 163.9, 157.4, 135.3 (4C), 134.8, 132.8, 131.5, 131.1, 130.5, 129.8, 128.8, 128.2 (4C), 127.7, 126.1, 125.4, 122.8, 115.5, 114.4, 40.1, 30.2, 26.6 (3C), 20.4, 19.7, 13.8 ppm. HRMS (ESI, shown in Figure S3): Calc. for C_32_H_33_N_1_O_3_Si_1_ [M + H]^+^ 508. 2302; Found: 508.2324.

## 3. Results and Discussion

### 3.1. Interaction between **Z2** and F^−^

**Figure 1.**
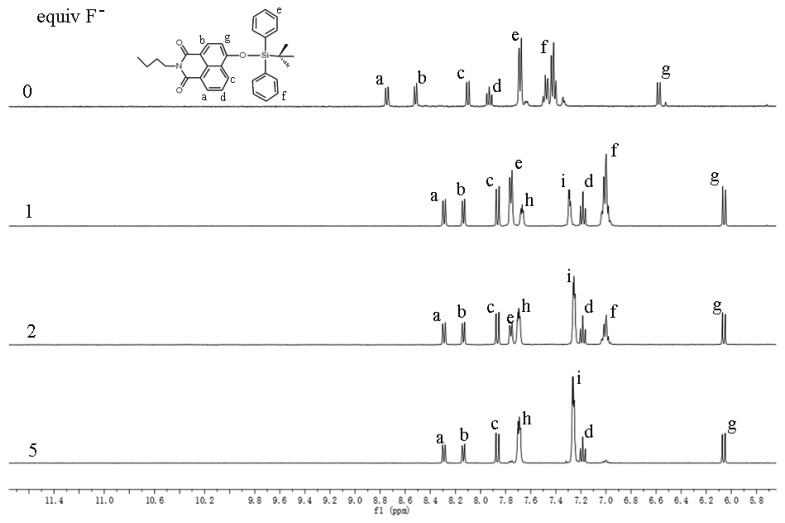
^1^H-NMR titration spectra of **Z2** (1.0 mM) with F^−^ ion (0, 1, 2, and 5 equivalents) in DMSO-d_6_.

**Scheme 1.**
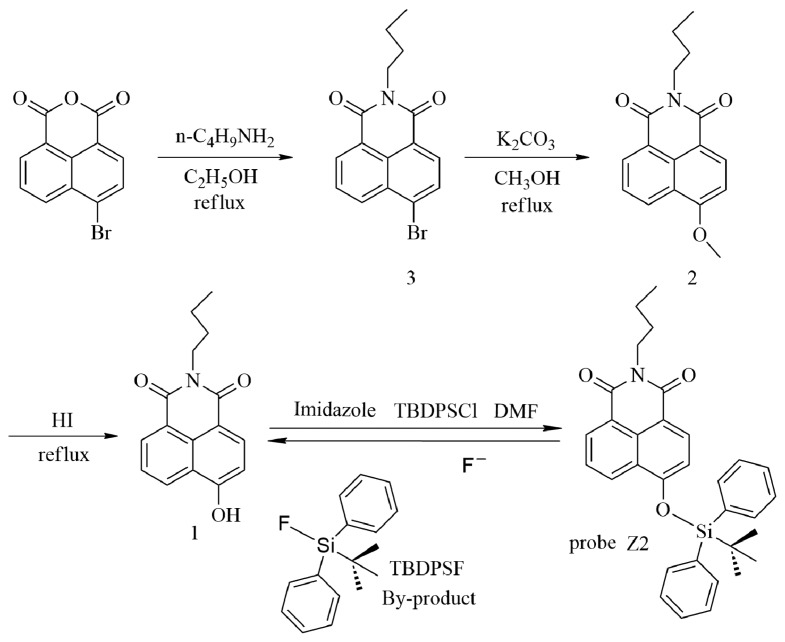
Synthetic route of **Z2** and the proposed mechanism of its response to F^−^.

To understand the mechanism of the interaction between **Z2** and F^−^, NMR titration was carried out (Figure 1). Addition of F^−^ ion resulted in the proton peaks of the naphthalimides moiety (“a–d” and “g”) upfield shifting, and the proton peaks “e” and “f”, which belonged to the TBDPS moiety, decreasing and disappeared at last. Meanwhile two new proton peaks (“h” and “i”) reflected the by-product TBDPSF appeared and elevated along with the increased concentrations of F^−^. This result indicated the reaction of **Z2** and F^−^ proceeded with the proposed mechanism in Scheme 1, which was in accordance with that presented in a previous paper [40].

### 3.2. Photophysical Property of **Z2**

**Figure 2.**
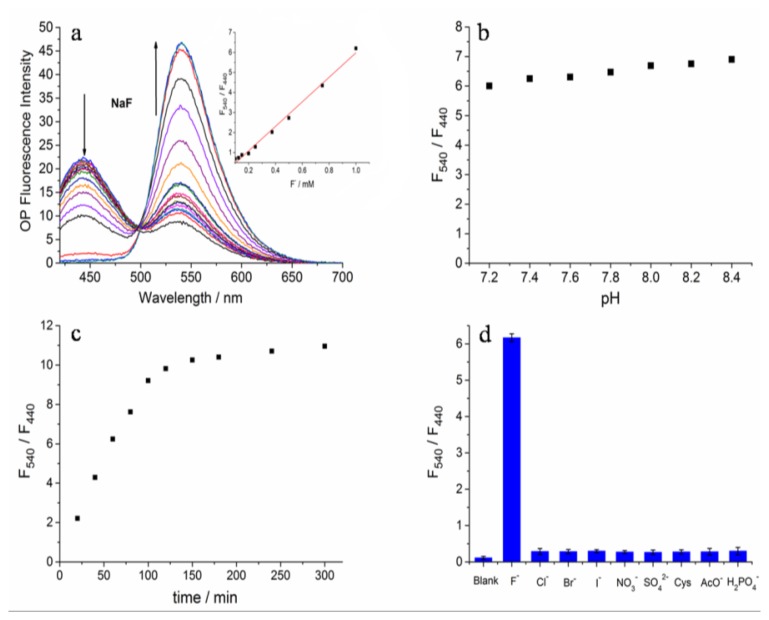
(**a**) OP Fluorescence spectra change of **Z2** toward different concentrations of F^−^ (0, 5, 10, 20, 40, 60, 80 100, 125, 150, 200, 250, 375, 500, 750, 1000, 1500 μM); (**b**) Effect of pH on fluorescence responses of **Z2**; (**c**) Fluorescence change of **Z2** with the reaction time; (**d**) Fluorescence responses of **Z2** toward F^−^ and other analytes (1 mM). λex = 410 nm; **Z2**: 5 μM; 20 mM HEPES and 30% DMSO; pH 7.4 except for (b); F^−^: 1 mM except (a).

The photophysical properties of **Z2** were studied systematically in HEPES buffer (20 mM, pH 7.4) containing 30% DMSO (v/v). The UV-Vis absorption spectrum and fluorescence excitation spectrum of **Z2** were examined first (Figure S4). Under these conditions, **Z2** exhibited a strong response toward F^−^. Upon adding F^−^, the absorption maxima at 365 nm decreased and a new band at 456 nm appeared (Figure S5). In the fluorescence measurements a new fluorescence maxima at 540 nm increased along with the disappearance of the fluorescence emission peak at 440 nm (Figure 2a). Moreover, a good linear relationship of F_540_/F_450_ (regression factor, R = 0.9858) with the concentration of F^−^ (0.1–1.0 mM) was observed (Figure 2a inset). The fluorescence emission could achieve stability in 150 min after adding F^−^ (Figure 2c). In addition, **Z2** showed negligible fluorescence responses toward pH changes (Figure 2b), which demonstrated **Z2** was insensitive to biologically relevant pH values. In order to evaluate the selectivity of **Z2** toward F^−^, the ions of Cl^−^, Br^−^, I^−^, NO_3_^−^, SO_4_^2−^, H_2_PO_4_^−^, AcO^−^ and cysteine (Cys) (1 mM each) were added separately. Only F^−^ induced unique fluorescence changes, and the others did not cause any fluorescence emission change (Figure 2d). Therefore, **Z2** was highly selective for F^−^ and could be employed to monitor F^−^ without interference from the other analytes mentioned above. The results were similar with those in a just published paper, in which the authors used different synthesis conditions to get the same product. They studied only OP fluorescence properties and the recognition capacity in 90% DMSO solution [43].

**Figure 3.**
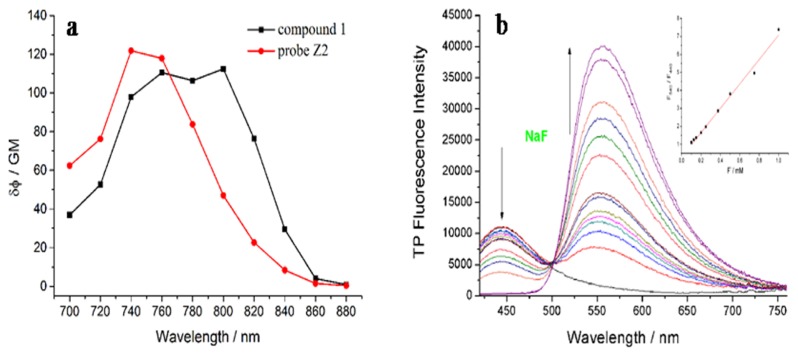
(**a**) Two-photon action cross section of **Z2** (red) and **1** (blank); (**b**) TP Fluorescence spectra change of **Z2** toward F^−^ with different concentrations (0, 5, 10, 20, 40, 60, 80, 100, 125, 150, 200, 250, 375, 500, 750, 1000, 1500 μM). **Z2**: 5μM; 20 mM HEPES pH 7.4, 30% DMSO; λex = 780 nm.

The TP absorption cross-section intensity of **Z2** and **1** were evaluated, respectively. As shown in Figure 3a, the maximum TP action cross section value of **Z2** was 121 GM at around 720 nm, and 112 GM at 800 nm after adding F^−^. Furthermore, the dose-dependent ratio F_540_/F_440_ of **Z2** displayed a good linearity for F^−^ (regression factor, R = 0.9878) with the concentration (0.1–1.0 mM), which predicted the probe could be used to detect F^−^ quantitatively by TP excitation (Figure 3b).

### 3.3. Imaging F^−^ in Live Cells and Deep Tissues

**Figure 4.**
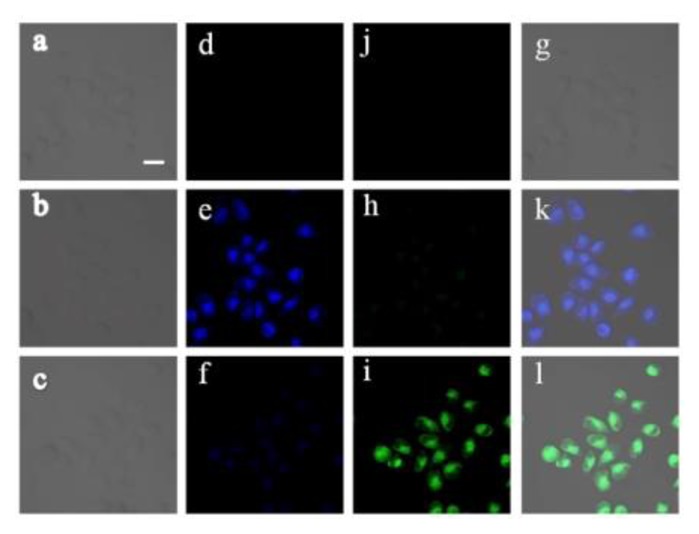
TPM fluorescence imaging of HeLa cells. **a**, **d**, **j**, **g**: Blank HeLa cells; **b**, **e**, **h**, **k**: Incubated with Z2 (10 μM) for 20 min; **c**, **f**, **i**, **l**: Incubated with Z2 (10 μM) for 20 min and then treated with F^−^ (15 μM) for another 20 min; **a**, **b**, **c**: Bright-field image; **d**, **e**, **f**: Blue channel at 400–450 nm; **j**, **h**, **i**: Green channel at 500–550 nm; **g**, **k**, **l**: Overlap channels. The scale bar is 20 μm.

To demonstrate the ability of **Z2** to image F^−^ in live cells, HeLa cells were used as the model cells. After cells incubated with **Z2** (10 μM) for 20 min, bright blue fluorescence could be observed inside cells (Figure 4e) upon excitation at 780 nm. After 15 μM of NaF was added to the above cells, strong green fluorescence appeared (Figure 4i). The distinct changes of fluorescence responses clearly indicated that **Z2** was capable of ratiometric fluorescence imaging of F^−^ in living cells.

Next, we investigated the utility of **Z2** in deep tissue imaging. TPM images were obtained from a part of a tumor tissue slice. The Z-scanning confocal imaging revealed that bright TP fluorescence emission was still present at 250 μm of penetration depth and no auto-fluorescence signal existed. The TPM images at depths of 50, 100, 150, 200, and 250 μm showed the F^−^ distribution in each XY plane.

**Figure 5.**
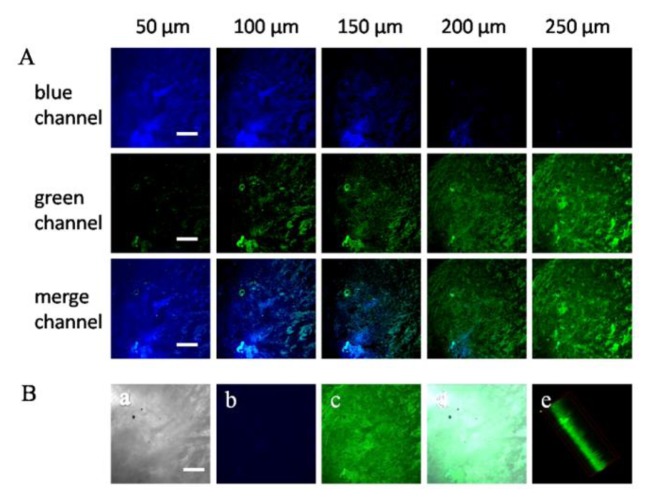
The confocal fluorescence imaging of a part of a fresh tumor tissue slice stained by **Z2** (15 μM). (**A**) Z-scan TP fluorescence images of **Z2** at different penetration depths using different channels; (**B**) Bright-field image of the tissue (**a**), TP fluorescence image after adding **Z2** at blue channel of 400–450 nm (**b**), and at green channel of 500–550 nm (**c**), overlay images of bright-field and green channel (**d**), the 3D reconstruction from 50 confocal Z-scan TPE imaging sections at depth of 0–500 μm with 60 × magnification (**e**). The scale bar was 60 μm.

These results indicated that **Z2** was able to ratiometrically detect F^−^ at the depths of 50–250 μm in tissues using TPM (Figure 5A). Then the 3D reconstitution of confocal XYZ scanning micrographs was obtained from 50 confocal Z-scan TPE imaging sections at depth of 0–500 μm (Figure 5B), which demonstrated that the **Z2** was evenly distributed in the tumor tissue and achieved a high signal-to-noise ratio between the tissue and the background.

## 4. Conclusions

In summary, we have developed a reaction-based TP ratiometric fluorescence **Z2**, which displayed significant two-photon action cross-section, good photostability, insensitivity toward biologically pH changes, high selectivity for fluoride ion and a marked ratiometric fluorescence emission change (100 nm). Moreover, **Z2** was successfully applied to ratiometric imaging of fluoride ion both in live cells and tissues at 50–250 μm depth. The results suggested that **Z2** could act as a valuable tool for monitoring fluoride ion in the complicated physiological environment, and might find use in medical research and clinical diagnostics.

## Supplementary Material



## References

[1] Newbrun E. (2010). What we know and do not know about fluoride. J. Public Health Dent..

[2] Black C.B., Andrioletti B., Try A.C., Ruiperez C., Sessler J.L. (1999). Dipyrrolylquinoxalines: Efficient sensors for fluoride anion in organic solution. J. Am. Chem. Soc..

[3] Beer P.D., Gale P.A. (2001). Anion recognition and sensing: The state of the art and future perspectives. Angew. Chem. Int. Ed..

[4] Horowitz H.S. (2003). The 2001 CDC recommendations for using fluoride to prevent and control dental caries in the united states. J. Public Health Dent..

[5] Dambacher M.A., Ittner J., Ruegsegger P. (1986). Long-term fluoride therapy of postmenopausal osteoporosis. Bone.

[6] Farley J.R., Wergedal J.E., Baylink D.J. (1983). Fluoride directly stimulates proliferation and alkaline phosphatase activity of bone-forming cells. Science.

[7] Holland M.A., Kozlowski L.M. (1986). Clinical features and management of cyanide poisoning. Clin. Pharm..

[8] Matsui H., Morimoto M., Horimoto K., Nishimura Y. (2007). Some characteristics of fluoride-induced cell death in rat thymocytes: Cytotoxicity of sodium fluoride. Toxicol. Vitr..

[9] Kleerekoper M. (1998). The role of fluoride in the prevention of osteoporosis. Endocrinol. Metab. Clin. North Am..

[10] Singh P., Barjatiya M., Dhing S., Bhatnagar R., Kothari S., Dhar V. (2001). Evidence suggesting that high intake of fluoride provokes nephrolithiasis in tribal population. Urol. Res..

[11] Cittanova M.L., Lelongt B., Verpont M.C. (1996). Fluoride ion toxicity in human kidney collecting duct cells. Anesthesiology.

[12] Bassin E.B., Wypij D., Davis R.B. (2006). Age-specific fluoride exposure in drinking water and osteosarcoma (United States). Cancer Causes Control.

[13] Cheng T.J., Chen T.M., Chen C.H., Lai Y.K. (1998). Induction of stress response and differential expression of 70 kDa stress proteins by sodium fluoride in HeLa and rat brain tumor 9L cells. J. Cell. Biochem..

[14] Barbier O., Arreola M.L., del Razo L.M. (2010). Molecular mechanisms of fluoride toxicity. Chem. Biol. Interact..

[15] Hutchinson J.P., Evenhuis C.J., Johns C., Kazarian A.A., Breadmore M.C., Macka M., Hilder E.F., Guijt R.M., Dicinoski G.W., Haddad P.R. (2007). Identification of inorganic improvised explosive devices by analysis of postblast residues using portable capillary electrophoresis instrumentation and indirect photometric detection with a light-emitting diode. Anal. Chem..

[16] Breadmore M.C., Palmer A.S., Curran M., Macka M., Avdalovic N., Haddad P.R. (2002). On-column ion-exchange preconcentration of inorganic anions in open tubular capillary electrochromatography with elution using transient-isotachophoretic gradients. 3. Implementation and method development. Anal. Chem..

[17] Marco R.D., Clarke G., Pejcic B. (2007). Ion-selective electrode potentiometry in environmental analysis. Electroanalysis.

[18] Van den Hoop M.A., Cleven R.F., van Staden J.J., Neele J. (1996). Analysis of fluoride in rain water comparison of capillary electrophoresis with ion chromatography and ion-selective electrode potentiometry. J. Chromatogr. A..

[19] Zhu B., Yuan F., Li R., Li Y., Wei Q., Ma Z., Zhang X. (2011). A highly selective colorimetric and ratiometric fluorescent chemodosimeter for imaging fluoride ions in living cells. Chem. Commun..

[20] Zhou Y., Zhang J.F., Yoon J. (2014). Fluorescence and colorimetric chemosensors for fluoride-ion detections. Chem. Rev..

[21] Song K.C., Kim H., Lee K.M., Lee Y.S., Do Y., Lee M.H. (2013). Ratiometric fluorescence sensing of fluoride ions by triarylborane-phenanthroimidazole conjugates. Sens. Actuators B.

[22] Hu R., Feng J., Hu D., Wang S., Li S., Li Y., Yang G. (2010). A rapid aqueous fluoride ion sensor with dual output modes. Angew. Chem. Int. Ed..

[23] Kim S.Y., Park J., Koh M., Park S.B., Hong J.I. (2009). Fluorescent probe for detection of fluoride in water and bioimaging in A549 human lung carcinoma cells. Chem. Commun..

[24] Li Y., Duan Y., Zheng J., Li J., Zhao W., Yang S., Yang R. (2013). Self-assembly of graphene oxide with a silyl-appended spiropyran dye for rapid and sensitive colorimetric detection of fluoride ions. Anal. Chem..

[25] Wang F., Wu J.S., Zhuang X.Q., Zhang W.J., Liu W.M., Wang P.F., Wu S.K. (2010). A highly selective fluorescent sensor for fluoride in aqueous solution based on the inhibition of excited-state intramolecular proton transfer. Sens. Actuators B.

[26] Zheng X., Zhu W., Liu D., Ai H., Huang Y., Lu Z. (2014). Highly selective colorimetric/fluorometric dual-channel fluoride ion probe, and its capability of differentiating cancer cells. ACS Appl. Mater. Interfaces.

[27] Khanmohammadi H., Rezaeian K. (2014). Naked-eye detection of inorganic fluoride in aqueous media using a new azo-azomethine colorimetric receptor enhanced by electron withdrawing groups. RSC Adv..

[28] Chen J.S., Zhou P.W., Zhao L., Chu T.S. A. (2014). DFT/TDDFT study of the excited state intramolecular proton transfer based sensing mechanism for the aqueous fluoride chemosensor BTTPB. RSC Adv..

[29] Roy A., Kand D., Saha T., Talukdar P. (2014). Pink fluorescence emitting fluoride ion sensor: Investigation of the cascade sensing mechanism and bioimaging applications. RSC Adv..

[30] Cao D.X., Liu Z.Q., Li G.Z. (2008). A trivalent organoboron compound as one and two-photon fluorescent chemosensor for fluoride anion. Sens. Actuators B.

[31] Yang S., Liu Y., Feng G. (2013). Rapid and selective detection of fluoride in aqueous solution by a new hemicyanine-based colorimetric and fluorescent chemodosimeter. RSC Adv..

[32] Das S.K., Lim C.S., Yang S.Y., Han J.H., Cho B.R. (2012). A small molecule two-photon probe for hydrogen sulfide in live tissues. Chem. Commun..

[33] Sun W., Fan J., Hu C., Cao J., Zhang H., Xiong X., Peng X. (2013). A two-photon fluorescent probe with near-infrared emission for hydrogen sulfide imaging in biosystems. Chem. Commun..

[34] Kang D.E., Lim C.S., Kim J.Y., Kim E.S., Chun H.J., Cho B.R. (2014). Two-Photon Probe for Cu^2+^ with an Internal Reference: Quantitative Estimation of Cu^2+^ in Human Tissues by Two-Photon Microscopy. Anal. Chem..

[35] Yan H., He L., Ma C., Li J., Yang J., Yang R., Tan W. (2014). Poly β-cyclodextrin inclusion-induced formation of two-photon fluorescent nanomicelles for biomedical imaging. Chem. Commun..

[36] Bae S.K., Heo C.H., Choi D.J., Sen D., Joe E.H., Cho B.R., Kim H.M. (2013). A Ratiometric Two-Photon Fluorescent Probe Reveals Reduction in Mitochondrial H_2_S Production in Parkinson's Disease Gene Knockout Astrocytes. J. Am. Chem. Soc..

[37] Lee H.W., Heo C.H., Sen D., Byun H.O., Kwak I.H., Yoon G., Kim H.M. (2014). A Ratiometric Two-Photon Fluorescent Probe for Quantitative Detection of β-Galactosidase Activity in Senescent Cells. Anal. Chem..

[38] Kim H.J., Heo C.H., Kim H.M. (2013). Benzimidazole-Based Ratiometric Two-Photon Fluorescent Probes for Acidic pH in Live Cells and Tissues. J. Am. Chem. Soc..

[39] Kim D., Singha S., Wang T., Seo E., Lee J.H., Lee S.J., Ahn K.H. (2012). *In vivo* two-photon fluorescent imaging of fluoride with a desilylation-based reactive probe. Chem. Commun..

[40] Zhang J.F., Lim C.S., Bhuniya S., Cho B.R., Kim J.S. (2011). A HighlySelective Colorimetric and Ratiometric Two-Photon Fluorescent Probe for Fluoride Ion Detection. Org. Lett..

[41] Albota M.A., Xu C., Webb W.W. (1998). Two-photon fluorescence excitation cross sections of biomolecular probes from 690 to 960 nm. Appl. Opt..

[42] Makarov N. S., Drobizhev M., Rebane A. (2008). Two-photon absorption standards in the 550–1600 nm excitation wavelength range. Opt. Express.

[43] Kai Y., Hu Y., Wang K., Zhi W., Liang M., Yang W. (2014). A highly selective colorimetric and ratiometric fluorescent chemodosimeter for detection of fluoride ions based on 1,8-naphthalimide derivatives. Spectrochim. Acta Part A.

