# Supplementary material for: Imaging of Fluoride Ion in Living Cells and Tissues with a Two-Photon Ratiometric Fluorescence Probe
Source: Sensors (Basel). 2015 Jan 14;15(1):1611–22. doi: 10.3390/s150101611 (PMC4327094; doi:10.3390/s150101611)
Supplement: Supplementary file 1 [file sensors-15-01611-s001.pdf]

OPEN ACCESS
sensors
ISSN 1424-8220
www.mdpi.com/journal/sensors

Supplementary Information

Imaging of Fluoride Ion in Living Cells and Tissues with a Two-Photon Ratiometric Fluorescence Probe. *Sensors* 2015, *15*, 1611-1622

Xinyue Zhu [1], Jianxi Wang [1], Jianjian Zhang [1], Zhenjie Chen [2], Haixia Zhang [1,*] and Xiaoyu Zhang [2]

[1] State Key Laboratory of Applied Organic Chemistry, College of Chemistry and Chemical Engineering, Lanzhou University, Lanzhou 730000, China.; E-Mails: zhuxy12@lzu.edu.cn (X.Z.); wangjx13@lzu.edu.cn (J.W.); zhangjj13@lzu.edu.cn (J.Z.)

[2] Institute of Physiology, School of Basic Medical Sciences, Lanzhou University, Lanzhou 730000, China. E-Mails: chenzj12@lzu.edu.cn (Z.C.); zhangxyu@lzu.edu.cn (X.Z)

* Author to whom correspondence should be addressed; E-Mail: zhanghx@lzu.edu.cn; Tel.: +86-931-8912510; Fax: +86-931-8912582.

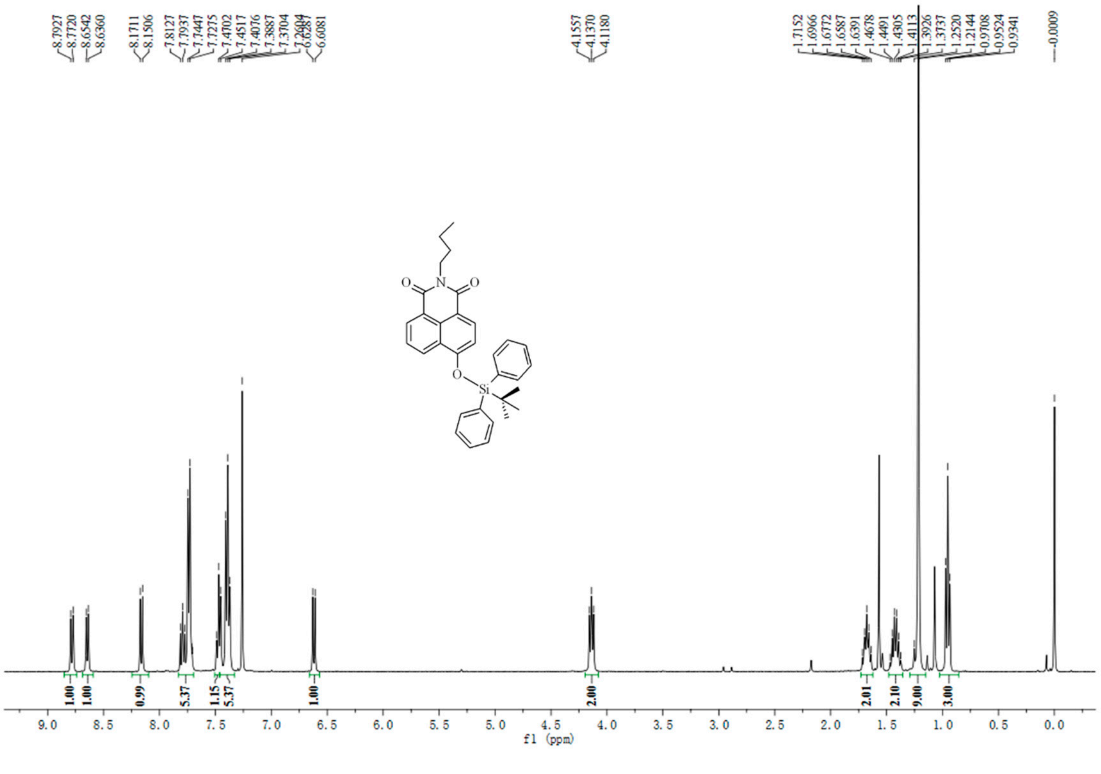

Figure S1. ^{1}H-NMR spectrum of probe Z2 in $CDCl_3$.

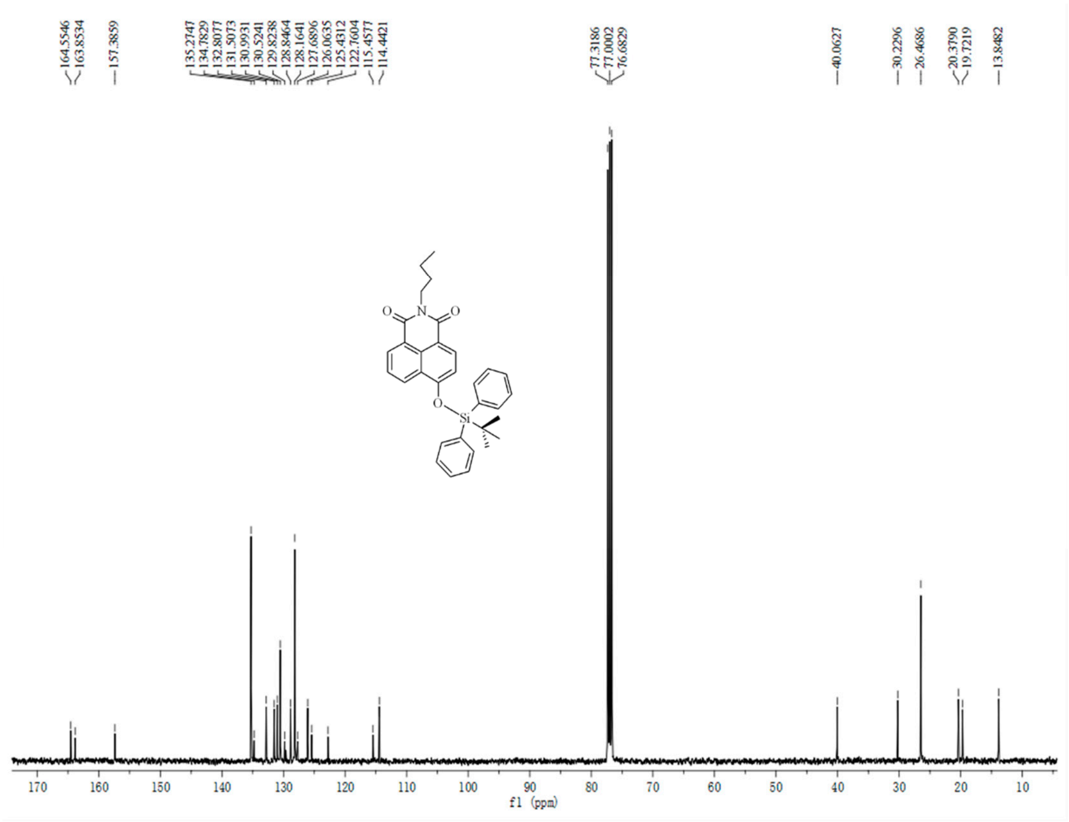

Figure S2. ^{13}C-NMR spectrum of probe Z2 in $CDCl_3$.

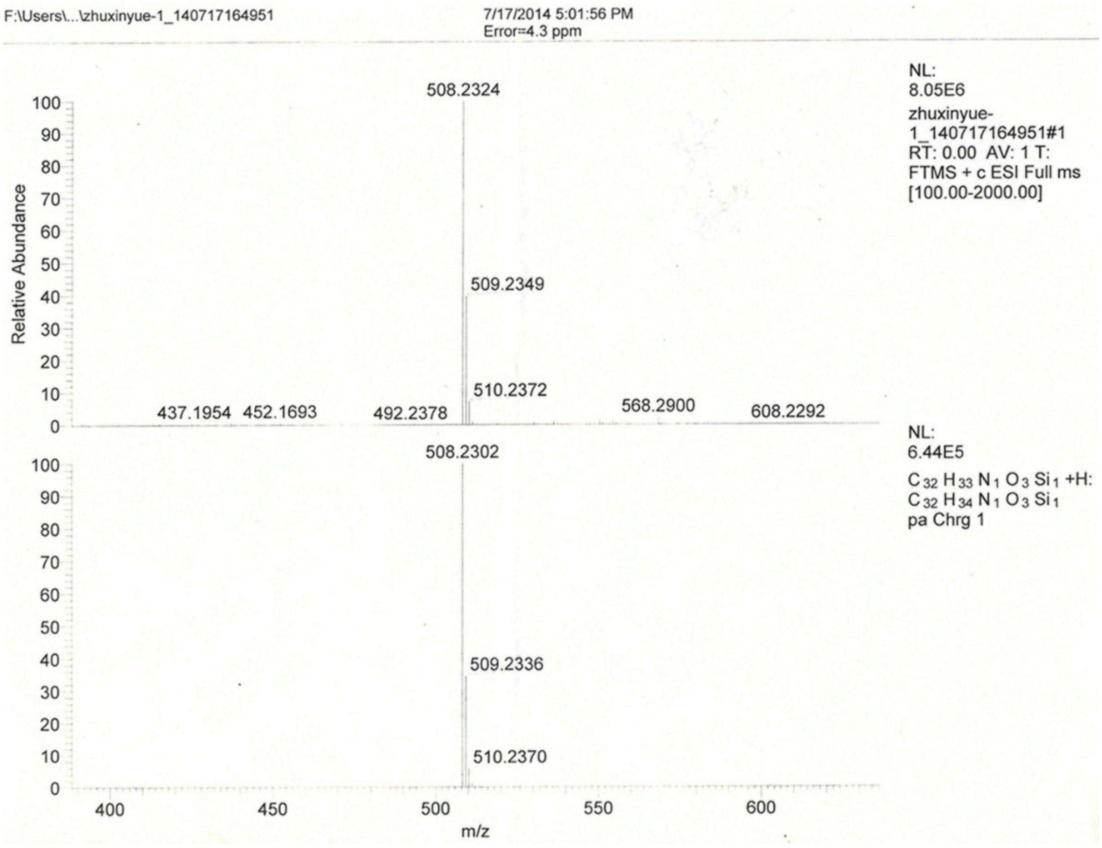

Figure S3. HRMS spectrum of probe Z2.

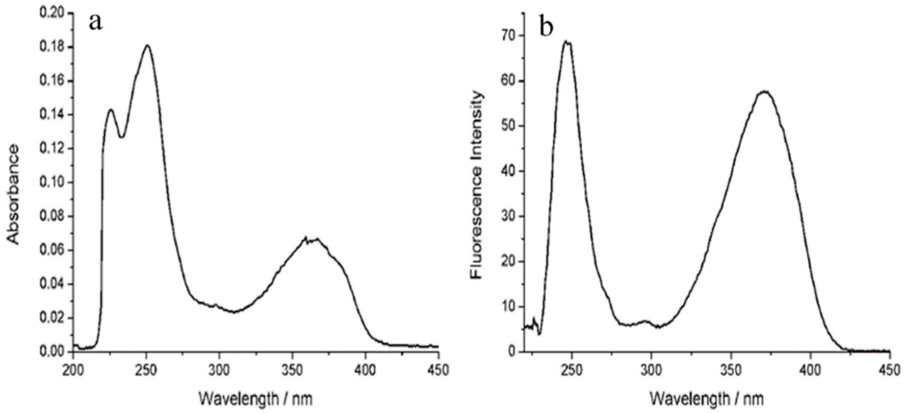

Figure S4. UV-Vis absorption spectrum (**a**) and fluorescence excitation spectrum (**b**) of probe Z2. (probe: 5 μM, HEPES, 20 mM pH 7.4, 30% ethanol).

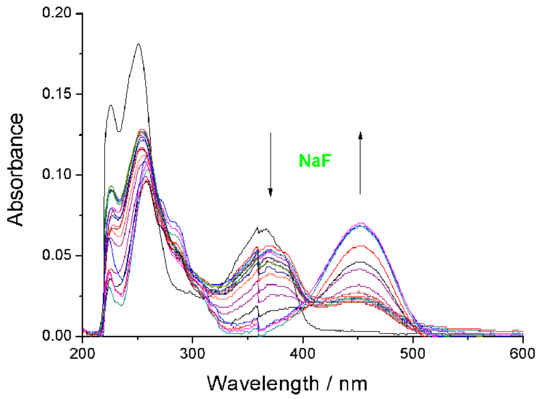

Figure S5. UV-Vis absorption spectra change of probe Z2 toward different concentrations F^{-} (0, 5, 10, 20, 40, 60, 80 100, 125, 150, 200, 250, 375, 500, 750, 1000, 1500 μM). (probe: 5 μM, HEPES, 20 mM pH 7.4, 30% ethanol).

© 2015 by the authors; licensee MDPI, Basel, Switzerland. This article is an open access article distributed under the terms and conditions of the Creative Commons Attribution license (http://creativecommons.org/licenses/by/4.0/).
